# Domestic Science

**Published:** 1889-03

**Authors:** 


### DOMESTIC SCIENCE.

### CHEMICAL CHANGES IN THE COOKING AND DIGESTION OF FOOD.

The demonstration lesson at the School of Domestic Science at the Young Women's Christian Association this morning was ably conducted by Mrs. Green of Concord, N. H. The subject under consideration was the "Chemistry of Food." Mrs. Green's introductory remarks were substantially as follows: Our bodies and our foods consist of essentially the same kind of material. The actually nutritious ingredients of our food may be divided into four classes: protein, fats, carbohydrates, and mineral matter. Leaving water out of account, lean meat, white of egg, curd of milk, and gluten of wheat consist mainly of protein compounds. Butter and lard are mostly fats. Sugar and starch are carbohydrates.

The nutrients of animal food consist mainly of protein and fats. Those of the vegetable foods are largely carbohydrates. The fatter kinds of meat and some species of fish, as salmon, shad or mackerel, contain considerable quantities of fat. The lean kinds of meat, and such fish, as cod and haddock, contain very little fat. Beans, peas, oatmeal and some other vegetable foods contain considerable quantities of protein.

The different nutrients have different offices to perform in the nutrition of the body. The demands of different people for nourishment vary with age, sex, occupation and other conditions of life. Health and pecuniary economy alike require that the diet should contain nutrients proportionate to the wants of the tissues.

Cheese is the most nourishing of any article of food. It contains all the properties necessary to the support of the body. One pound of cheese is worth three pounds of beefsteak. Next in order of nutrition come peas, and then beans. Two and one half pounds of potatoes are equal in carbon to one pound of bread, and 3½ pounds of potatoes are equal in nutrition to one pound of bread. The reason that so many people in Ireland live and thrive so well upon potatoes, is because they eat so much buttermilk with them. The buttermilk contains what the potato lacks in nutrition.

The speaker then proceeded to prepare a dish that contained everything necessary for the support of the body.

The dish is prepared as follows ; Take $\frac{1}{4}$ pound macaroni ; do not wash it, as it removes the starch ; put it into boiling water , add one tablespoon of salt ; keep boiling rapidly until done. When soft take it off the fire and pour cold water through it ; put it into a buttered dish, cut into pieces about six inches long, then pour a sauce over it which is made as follows : One cup and half of boiling milk, one tablespoon of butter, a heaping tablespoon of flour, $\frac{1}{2}$ teaspoon of salt, and a saltspoon of pepper ; stir the butter and flour together until they bubble ; stir in the salt and pepper, then add the milk gradually, after which pour over the macaroni. Take one half cup of grated cheese, sprinkle over the top ; over the cheese sprinkle one cup of cracker crumbs ; into the cracker crumbs put a saltspoon of pepper. Bake until the crumbs are brown in a quick oven.

Macaroni is acknowledged by all scientists to contain more gluten than bread. In all the long list of articles used for food there are only two perfect articles. They are milk and eggs.

